# A non-invasive method to assess environmental contamination with avian pathogens: beak and feather disease virus (BFDV) detection in nest boxes

**DOI:** 10.7717/peerj.9211

**Published:** 2020-06-11

**Authors:** Johanne M. Martens, Helena S. Stokes, Mathew L. Berg, Ken Walder, Shane R. Raidal, Michael J.L. Magrath, Andrew T.D. Bennett

**Affiliations:** 1 Centre for Integrative Ecology, School of Life and Environmental Sciences, Deakin University, Waurn Ponds, Victoria, Australia; 2 Centre for Molecular and Medical Research, School of Medicine, Deakin University, Waurn Ponds, Victoria, Australia; 3 School of Animal and Veterinary Sciences, Faculty of Science, Charles Sturt University, Wagga Wagga, New South Wales, Australia; 4 Wildlife Conservation and Science, Zoos Victoria, Parkville, Victoria, Australia

**Keywords:** BFDV, Fomites, Indirect transmission, Parrots, PBFD, Psittacine beak and feather disease, Reservoir, Spill-over, Nest box

## Abstract

Indirect transmission of pathogens can pose major risks to wildlife, yet the presence and persistence of wildlife pathogens in the environment has been little studied. Beak and feather disease virus (BFDV) is of global conservation concern: it can infect all members of the Psittaciformes, one of the most threatened bird orders, with infection often being lethal. Indirect transmission of BFDV through contaminated nest hollows has been proposed as a major infection source. However, data on whether and for how long nest sites in the wild remain contaminated have been absent. We determined the BFDV status of birds (parents and nestlings) for 82 nests of Crimson Rosellas, *Platycercus elegans* and Eastern Rosellas, *Platycercus eximius*. In 11 of these nests (13.4%, 95% confidence interval 6.9–22.7), we found an infected parent or nestling. Using nest swabs, we then compared BFDV presence at three points in time (before, during and after breeding) in three groups of nest boxes. These were nest boxes occupied by infected birds, and two control groups (nest boxes occupied by uninfected birds, and unoccupied nest boxes). Detection of BFDV on nest swabs was strongly associated with the infection status of parents in each nest box and with the timing of breeding. During breeding, boxes occupied by BFDV-positive birds were significantly more likely to have BFDV-positive nest swabs than boxes occupied by BFDV-negative birds; nest swabs tested BFDV-positive in 80% (28.4–99.5) of nests with parental antigen excretion, 66.7% (9.4–99.2) of nests occupied by parents with BFDV-positive cloacal swabs and 66.7% (22.3–95.7) of nests occupied by parents with BFDV–positive blood. 0% (0–52.2) of nests with BFDV–positive nestlings had BFDV–positive nest swabs. Across all boxes occupied by BFDV-positive birds (parents or nestlings), no nest swabs were BFDV–positive before breeding, 36.4% (95% CI 10.9–69.2) were positive during breeding and 9.1% (0.2–41.3) remained positive after breeding. BFDV was present on nest swabs for up to 3.7 months. Our study provides novel insights into the potential role of nest cavities and other fomites in indirect transmission of BFDV, and possibly other pathogens, and offers a non-invasive method for surveillance of pathogens in wild bird populations.

## Introduction

Infection with pathogens can occur not only through direct (host-host) transmission, but also through indirect (host-environment-host) transmission (Guan & Holley, 2003; Seymour & Appleton, 2001). Indirect transmission is highly dependent on the ability of a pathogen to survive outside of the host (Heaton & Jones, 2008). Some pathogens can persist for months (Nicholson, Groves & Chambers, 2005) or even years outside of the host, and thereby pose a threat of infection through contact with the pathogen-contaminated environment (Kramer, Schwebke & Kampf, 2006; Murray et al., 2016). In most wildlife, the role of environmental contamination and the resulting possible indirect transmission of pathogens are little studied, often because suitable non-invasive methods have not been developed (Hudson et al., 2002). Yet as shown by various modelling approaches, neglecting sources of indirect transmission can lead to considerably less accurate predictions of pathogen persistence, outbreak probability, outbreak duration (Rohani et al., 2009) and of the need for management efforts (Lange, Kramer-Schadt & Thulke, 2016). This is particularly concerning as many emerging diseases causing epidemics in humans originate in wildlife (Daszak, Cunningham & Hyatt, 2000), as demonstrated most recently in a novel coronavirus strain (Wu et al., 2020). Studies on suitable methodology and thus field data on detection of pathogens at potential fomites, such as nesting sites or feeding stations, could provide improved disease surveillance and hygiene protocols in species recovery programs.

Most studies on environmental contamination with pathogens have been conducted under laboratory conditions, with inoculated animals sacrificed for testing (Kallio et al., 2006; Palmer, Waters & Whipple, 2004). One such study on House Finches (*Haemorhous mexicanus*) infected with the bacterium *Mycoplasma gallisepticum* showed that the pathogen could be detected using swabs of the environment inhabited by hosts (Adelman et al., 2013). Vong et al. (2008) successfully used soil swabs to detect environmental contamination of human households with Influenza A virus from domestic poultry. However, this approach using swabs has not been applied for pathogen surveillance in wild bird populations, where it could potentially provide a useful, non-invasive method for surveillance of environmental contamination with pathogens, the results of which could help to reduce indirect transmission. To our knowledge, environmental contamination of nesting material with avian pathogens has rarely been investigated. One example is a study by Sikorski et al. (2013), who collected and tested the nesting material in a single nest.

Our goal was to test a surveillance method and elucidate the tractability of environmental sampling for non-invasive pathogen surveillance in wild populations, while simultaneously providing insight into the potential for indirect transmission of an avian virus causing serious conservation concern globally. We used swabs from nests of wild parrots as a non-invasive method for wildlife pathogen detection, using beak and feather disease virus (BFDV) in congeners Crimson Rosella (*Platycercus elegans*) and Eastern Rosella (*Platycercus eximius*) as our model system. BFDV is a generalist ssDNA circovirus (Sarker et al., 2014) which causes one of the most significant diseases of psittacine birds around the world, Psittacine Beak and Feather Disease (PBFD) (Fogell, Martin & Groombridge, 2016). All psittacines world-wide are considered susceptible (Fogell et al., 2018). Additionally, BFDV has recently been reported in several non-psittacine bird species (Amery-Gale et al., 2017; Sarker et al., 2016; Sarker et al., 2015), indicating susceptibility beyond parrots. BFDV is considered a threat in captive breeding programs for several endangered species (Raidal, Sarker & Peters, 2015). The peracute and acute forms of PBFD are usually observed in young nestlings (Perry, 1981), which typically die within one to two weeks after first appearance of clinical signs, or develop the chronic form of PBFD (Perry, 1981; Raidal, 1995). Given nestlings are particularly likely to become infected, and given BFDV has been considered stable in the environment (Raidal & Peters, 2018), it has been hypothesised that nest hollows may contribute significantly to BFDV transmission (Peters et al., 2014).

BFDV is thought to be transmitted directly between individuals through faeces, contaminated feather dust and crop secretions (Ritchie et al., 1991), as well as from mothers to embryonated eggs (Rahaus et al., 2008). It was found in mites infesting a BFDV-positive cockatoo, suggesting that it may be transmitted through ectoparasitic vectors (Portas et al., 2017). BFDV is considered highly stable in the environment (Raidal & Peters, 2018; Todd, 2000) because it is resistant to heat, surviving exposure to high temperatures, for example, 80 °C for 30 min (Raidal & Cross, 1994), and many disinfectants (Jackson et al., 2014). Consequently, indirect transmission through contaminated nest hollows is considered likely, as infected birds shed high amounts of BFDV in both feather dander and faeces into the environment (Raidal, Sabine & Cross, 1993). Additionally, Psittaciformes often compete for limited nest hollows (Heinsohn, Murphy & Legge, 2003), which may increase the potential for interspecies transmission through contaminated nest hollows (Peters et al., 2014). However, to our knowledge, no study has investigated the presence and persistence of BFDV in nesting material. Understanding the role of contaminated nest boxes in disease transmission and the potential impact they might have on wild, as well as managed, populations is crucial (Peters et al., 2014), and a key step in developing a method for decreasing rates of indirect transmission and improving pathogen management outcomes (Australian Department of Sustainability Environment, Water, Population and Communities, 2012). Such a method, which would allow simple, standardised sampling from nest boxes for BFDV detection, would aid targeted BFDV surveillance in species recovery programs, many of which do not yet include BFDV as a risk (Australian Department of the Environment and Heritage, 2005).

Here, we investigated BFDV presence and persistence in occupied and unoccupied nest boxes using paired controls and repeated measures over two breeding seasons. We determined BFDV prevalence and viral shedding of birds occupying nest boxes as part of a long-term study in breeding *P. elegans* and *P. eximius* and their offspring (Eastwood et al., 2019). Both species have been shown to be susceptible to BFDV infection, with 34.5% prevalence in wild *P. elegans* in Australia (Eastwood et al., 2015), subject to substantial variation with age and subspecies (Eastwood et al., 2014), and 14.8% prevalence in introduced *P. eximius* in New Zealand (Ha et al., 2007). In wild *P. elegans*, Eastwood et al. (2019) found that BFDV infection status was not correlated between parents and their offspring, as in some nest boxes, one or more nestlings were BFDV-positive, although their parents were BFDV-negative. This pattern suggests infection of nestlings by indirect transmission from contaminated nesting material. We had three main aims: (1) Test whether and for how long BFDV can be detected using nest box swabs, (2) Investigate whether detectability in swabs is related to occupation of the box, and whether birds breeding in the box are infected with or shedding BFDV, and (3) Investigate whether presence of BFDV in nest boxes changes across the breeding season before, during and after occupation by breeding birds. This study will be an important step towards a non-invasive method for pathogen surveillance in nest boxes, and will shed light on the dynamics of indirect transmission.

## Material and Methods

### Sampling nest boxes and birds

Our study was carried out under Deakin University animal ethics approval (B31-2015), Australian Bird and Bat Banding authority 2319 and complied with the laws of Victoria (research permit 10007969). We collected 1500 nest swabs from 241 nest boxes used by *P. elegans* and *P. eximius*, from 2016 to 2018. The boxes were located at four locations in Victoria, Australia: Bellbrae (38.33152°S, 144.18677°E), Gundrys Road (38.34407°S, 144.21313°E), Meredith (37.84567°S, 144.07718°E) and Steiglitz (37.87573°S, 144.17980°E). They were constructed of 19 mm thick treated pine (*Pinus radiata*) and were 24 cm wide, 28 cm deep and 42 cm high with an entrance diameter of 7.5 cm and a sliding side door to permit easy access by researchers (Berg & Ribot, 2008). When the nest boxes were erected, a layer of Red Gum (*Eucalyptus camaldulensis*) wood chips was put inside each box to a depth of about 10 cm (Larson et al., 2015).

We used the following standardised protocol. Nest swabs were collected from each nest box before, during and after each of two breeding seasons, between September 2016 and January 2018. The swabs used were sterile, individually wrapped cotton tip swabs with plastic handles (Westlab, Mitchell Park, Australia). We swabbed active nests, that is, nests containing eggs or nestlings. We also took swabs from paired controls, which were randomly assigned unoccupied nest boxes in the same field site as their paired active box. Swab samples were taken fortnightly during the breeding season, and monthly between breeding seasons. When sampling a nest box, the cotton tip swab was first pulled along all four nest box walls approximately five centimetres above the nesting material, a height at which both adult and juvenile *P. elegans* and *P. eximius* would frequently be in contact with the nest box walls. The same swab was then inserted at three random locations into the nesting material at the base of the box. The swab tip was then placed in an empty 1.5 ml Eppendorf Safe-Lock Tube (Eppendorf AG, Hamburg, Germany), stored at 4 °C, and then transferred to −80 °C storage within eight hours. In nest boxes that did not contain nesting material (as some breeding birds removed all the Red Gum wood chips), the same movements were performed with the swab, but only the wooden floor of the nest box could be swabbed.

We determined the BFDV status of parents and nestlings in 82 nests in which nestlings successfully hatched during the two breeding seasons we investigated (*n* = 68 *P. elegans*, *n* = 13 *P. eximius*, and *n* = 1 mixed species brood *P. elegans* and *P. eximius* (Martens et al., 2018)). Sample sizes of blood samples were as follows: 122 blood samples of parents, 352 of nestlings when they were approximately one week old and 227 when they were approximately four weeks old. We used the following protocol to collect blood from breeding birds and their nestlings in order to test them for BFDV. Parents were trapped in the nest box during nestling provisioning (Berg & Ribot, 2008) and blood samples (approximately 100 µl from the brachial vein) and cloacal swabs taken from each individual. Blood samples were also taken from nestlings at one and four weeks of age. Blood was stored in ethanol at room temperature, and cloacal swabs at 4 °C in the field, then frozen at −80 °C upon return to the laboratory on the same day. To avoid virus transmission between sampled birds, birds were handled using nitrile gloves, the cotton bags used to hold birds in were autoclaved after each use, blood sampling equipment was single-use and banding and measuring tools were sprayed with F10 SC Veterinary Disinfectant (Health and Hygiene Pty Ltd, South Africa) after each use. For both parent and nestling samples, BFDV presence was determined using a quantitative real-time PCR (qPCR) assay (Eastwood et al., 2015). Antigen excretion of parents that had BFDV-positive blood (in our study, females only) as detected by qPCR was determined by testing chest feathers with a haemagglutination (HA) assay (Raidal, Sabine & Cross, 1993). We sampled one or more birds at 82 nests; nests where eggs did not hatch were excluded from this study, as we could not sample birds in these nests. *P. elegans* were aged (subadult (<1 year), young adult (1–3 years) and adult (>3 years)) based on distinct plumage colouration (Eastwood et al., 2017). This was not possible for *P. eximius*.

### Sampling design

**Figure 1:**
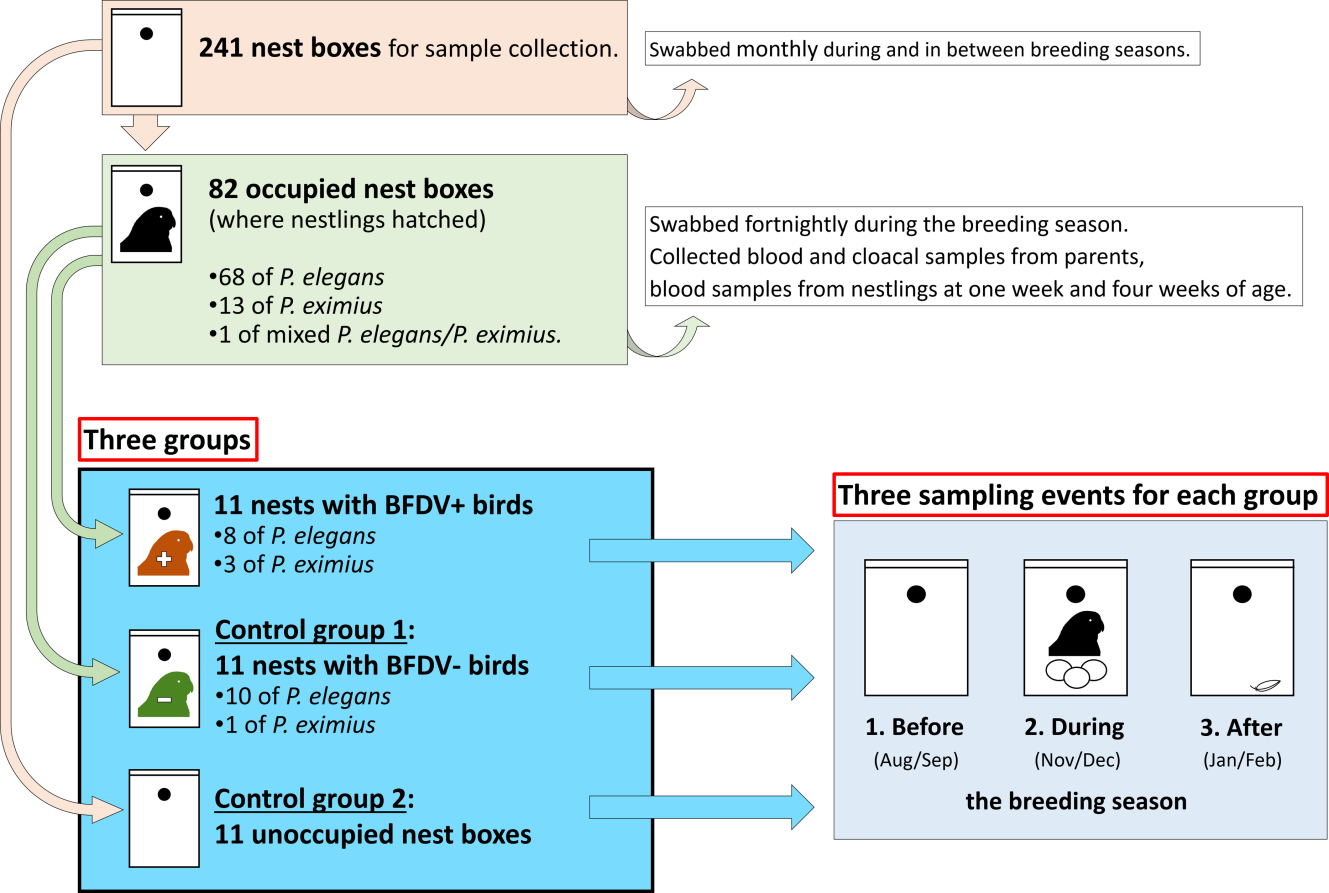
Experimental design. We tested nest swabs collected from three test groups of nest boxes before, during and after the breeding season. Nest boxes were assigned to their respective group depending on their status during the breeding season, i.e. if they were unoccupied, or occupied by breeding * P. elegans* or * P. eximius* and their nestlings, and if any of the birds (parents or nestlings) occupying the nest box were BFDV-positive or BFDV-negative. Nest boxes for control group 1 were chosen randomly from boxes containing nests where we had complete BFDV data of blood and cloacal swabs for both parents, and complete blood data for all nestlings, and all these samples were BFDV-negative as determined by qPCR. Nest boxes for control group 2 had been selected as paired controls for active nests during the breeding season. Each of the three test groups consisted of 11 nest boxes. ‘BFDV+’ stands for BFDV-positive, ‘BFDV-‘ stands for BFDV-negative. ‘Birds’ refers to parents and/or nestlings.

We subsampled our nest swabs, which had been taken at monthly intervals during the year and fortnightly during the breeding season, in order to create three categories of nest swabs: ‘before’, ‘during’ and ‘after’ breeding (Fig. 1). Swabs for the category ‘before’ were collected in August and September, before we found any nests in the boxes. Swabs for the category ‘during’ were collected from nests containing nestlings and paired control nest boxes fortnightly during the period when breeding was taking place in our study population (October–January). Of these, we tested the swabs taken closest to the date (range: 0–8 days before or after) when BFDV-positive blood or cloacal swab samples were collected from birds, mostly during late November and early December. For the category ‘after’, swabs were collected after the end of the breeding season, in late January and early February.

For nest boxes that contained infected (hereafter ‘BFDV-positive’) birds, equal numbers of ‘control boxes’ (Fig. 1) were selected: ‘control group 1’ contained nest boxes occupied by non-infected (hereafter ‘BFDV-negative’) birds (Table S2). These boxes were chosen randomly from boxes containing nests where we had complete BFDV data of blood and cloacal swabs for both parents, and complete blood data for all nestlings, and all these samples were BFDV-negative as determined by qPCR. ‘Control group 2’ consisted of nest boxes unoccupied by breeding birds in the years we conducted this study, and which had been selected as paired controls for active nests during the breeding season. For unoccupied nest boxes during the breeding season the sample size was 10 instead of 11, as one box was occupied by Sugar Gliders (*Petaurus breviceps*) for several weeks and could not be swabbed during this period. Nest boxes of both control groups were at the same field sites, so were located in the same habitats as the nest boxes containing BFDV-positive birds.

### DNA extraction & BFDV detection

To extract DNA from swab samples, we used an ammonium acetate DNA protocol (Bruford et al., 1998; Eastwood et al., 2015) which we modified for swabs. Modifications were as follows: swabs were heated to 56 °C for five minutes to inactivate potential zoonotic pathogens (Rush & Timms, 1996). Swabs were then transferred into fresh tubes containing 250 µl Digsol buffer, and 10 µl Proteinase K were added, followed by an overnight incubation at 37 °C. Then, 300 µl 4M ammonium acetate was added, and samples were centrifuged at 18k g for 25 min. The resulting supernatant was transferred into a fresh tube and centrifuged for 10 min to remove the last remains of swab cotton wool. 1 ml of 100% ethanol was added, and the mixture centrifuged for 15 min. The 100% ethanol was removed and 500 µl of 70% ethanol added to rinse the DNA pellet, then the mix was centrifuged for 10 min. The ethanol was then aspirated and the DNA pellets were left to dry by leaving the Eppendorf tubes upright and open, covered with sterile wipes, in a 25 °C heat block for three hours. Then 100 µl TE buffer was added and the samples were frozen at −20 °C until analysis. We ran extraction controls for a subset of 92 blood samples. None of these extraction controls tested BFDV-positive, suggesting that no lab contamination occurred (see Discussion). The extractions for blood samples, nest swabs and cloacal swabs were done by the same two persons, in the same lab and under the same conditions, so we do not think it is likely that a significant level of lab contamination occurred. Initial trials revealed possible inhibitors in the DNA solution, as none of the nest swabs in these trials, including a sample with added BFDV DNA, tested as BFDV-positive. The extracted DNA samples were then cleaned by spinning them in Zymo OneStep Inhibitor Removal columns (Zymo Research, Irvine, USA) for subsequent analyses.

For BFDV detection in nest swabs, we used the same probe-based qPCR method as for BFDV detection in blood and cloacal swab samples (Eastwood et al., 2015). The qPCR was performed using the PikoReal Real-Time PCR System (Thermo Fisher Scientific Inc., Waltham, USA). Positive controls (DNA of confirmed BFDV-positive *P. elegans* blood samples) and no-template controls were added to each qPCR plate, and all samples were run in duplicate. Samples that tested BFDV-positive in both wells were re-run to confirm BFDV presence. We set a conservative detection threshold (hereafter named ‘threshold_36_’) at Cq 36 based on an earlier study with the same method (Martens et al., 2019), meaning that we considered samples as BFDV-positive if they had Cq values (cycle at which probe fluorescence crosses the arbitrarily set detection baseline) of under 36 in both wells. Based on the repeatability of our results (qPCR results were repeatable up to a detection threshold of Cq 38 in the study presented here), we also tested a higher threshold of Cq 38 for nest swabs (hereafter named ‘threshold_38_’), meaning that weaker qPCR signals were counted as BFDV-positive.

When testing birds for BFDV, we first tested blood samples of all breeding birds and their nestlings, because reporting BFDV prevalence based on blood samples is a widely used and validated method for BFDV surveillance (Eastwood et al., 2015). We then tested cloacal swabs from parents that were BFDV-positive in blood samples, as well as from all other birds in the same nest as these infected birds, and from all birds in the control nests. For analysis of blood samples and cloacal swabs, we used threshold_36_ based on repeatability on qPCR results for these sample types (Martens et al., 2019).

### Statistical analyses

To compare differences in BFDV presence on nest swabs between the three test groups (nest swabs from before, during and after breeding), we used the Freeman-Halton extension of the Fisher exact probability test for a two-rows by three-columns contingency table. For pairwise comparisons of groups, we used the Fisher’s exact test with a 2x2 contingency table. All percentages are reported ± 95% confidence intervals unless otherwise stated.

## Results

### BFDV on nest swabs

**Figure 2:**
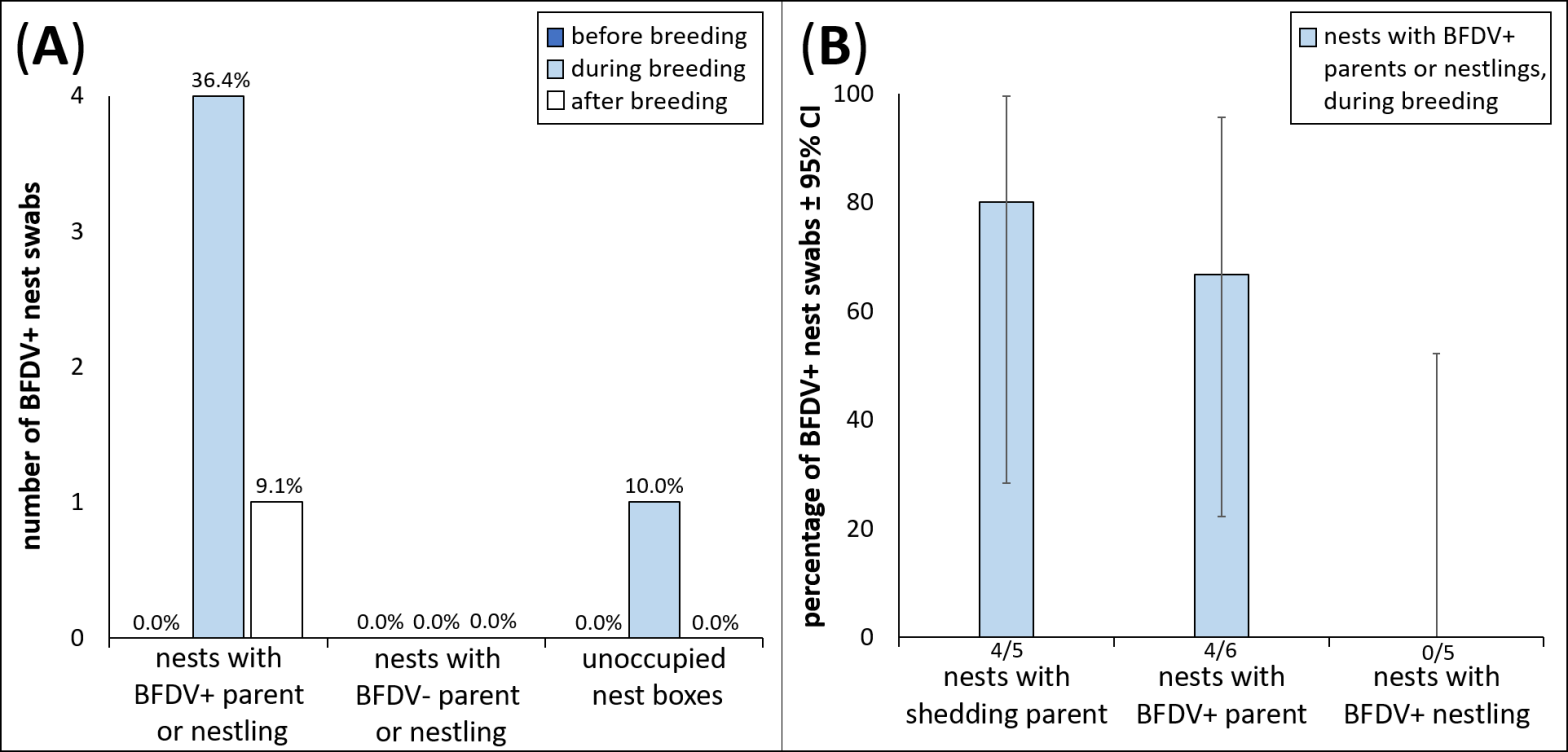
Number of nest boxes, in all three test groups, with BFDV-positive nest swabs before, during and after breeding, and percentage of BFDV-positive nest swabs in nests with BFDV-positive adults or nestlings, and BFDV shedding parents, during the breeding season. (A) number of nest boxes (out of total of 33 for the three test groups, 11 per group) with BFDV-positive nest box swabs before, during and after the breeding season, analysed with detection threshold _36_. For unoccupied nest boxes during the breeding season, the sample size was 10 instead of 11. (B) percentage of nest boxes with BFDV-positive (‘BFDV+’) nest swabs ± 95% confidence intervals, which were occupied by BFDV shedding parents (as detected with HA; only females could be tested for shedding, see Materials & Methods), by BFDV-positive parents, or by BFDV-positive nestlings (as detected with qPCR of blood samples), during the breeding season. Shedding parents are also BFDV-positive as detected by qPCR, and are also part of the bar showing ‘nests with BFDV+ parent’. The number at the base of bars indicates number of nest boxes with BFDV-positive nest box swabs out of the total number of nest boxes occupied by BFDV-positive or shedding parents or nestlings.

Of 82 nests tested, 11 nests (*n* = 8 *P. elegans*, *n* = 3 *P. eximius*) contained either one nestling or at least one parent which was BFDV-positive using the conservative threshold_36_ (11 of 82, 13.4%, 6.9–22.7; Table 1). We detected BFDV in six of 122 blood samples of parents (1.8%, 1.8–10.4), one of 352 (0.3%, 0.0–1.6) of one week old nestlings and four of 227 (1.8%, 0.5–4.5) of four week old nestlings. With one exception where both parents were BFDV-positive, we found only one infected bird (parent or nestling) in each BFDV-positive nest (Table 1). All parents that were BFDV-positive in blood were young females; the only male parent that was BFDV-positive in these nests had a BFDV-positive cloacal swab, but BFDV-negative blood (Table 1). In the remaining 71 nests, all parents and nestlings were BFDV-negative. For all 11 nest boxes occupied by a BFDV-positive parent or nestling (hereafter treated as one group, ‘BFDV-positive birds’), nest box swabs were BFDV-negative before breeding started. Four of the 11 (36.4%, 95% CI [10.9–69.2]) nests had BFDV-positive nest swabs during breeding, and one nest (9.1%, 95% CI [0.2–41.3]) was still BFDV-positive after the breeding season, using threshold_36_ (Fig. 2A, Fig. S1A). During breeding, BFDV-positive nest swabs were found in four of the six nests (66.7%, 95% CI [22.3–95.7]) containing parents with BFDV-positive blood samples, in two of the three nests (66.7%, 95% CI [22.3–95.7]) containing parents with BFDV-positive cloacal swabs, but in none of the five nests which contained BFDV-positive nestlings (0.0%, 95% CI [0.0–52.2], Table 1).

**Table 1:** Infection patterns in nest boxes which contained a BFDV-positive parent or nestling. In all nests in which we detected infected birds, only one bird per nest was BFDV-positive. White background indicates nests with BFDV-positive nest swabs; grey background indicates nests with BFDV-negative nest swabs. All nestlings were tested twice, at approximately one and four weeks of age. *P. elegans* and *P. elegans* nestlings stay in the nest for approximately five weeks from hatching to fledging (Higgins, 1999). BFDV-positive samples are marked as, BFDV-negative samples are marked as. Some sample types could not be tested for some of the birds (NT stands for not tested). HA titre shows levels of BFDV antigen excretion detected in feathers (only females could be tested). Age of infected nestling shows at what age approximately the BFDV-positive sample was taken.

**Nest box**	**Species**	**BFDV statusnest swab**	**HA titre BFDV+**♀**parent(log**_**2**_**)**	**BFDV status**♀**parent**		**BFDV status**♂**parent**		BFDV status nestlings	**Age of infected nestling**
				**blood**	**cloaca**	**blood**	**cloaca**	**blood**	
1	*P. elegans*	**+**	3	**+**	**+**	–	**+**	–	
2	*P. elegans*	**+**	6	**+**	**+**	–	NT	–	
3	*P. elegans*	**+**	6	**+**	–	–	–	–	
4	*P. elegans*	**+**	1	**+**	NT	–	–	–	
5	–	<1	**+**	**+**	–	–	–		
6	*P. eximius*	–	2	**+**	NT	NT	NT	–	
7	*P. elegans*	–		–	–	–	–	**+**	4 weeks
8	*P. elegans*	–		–	–	–	–	**+**	4 weeks
9	*P. elegans*	–		–	–	–	–	**+**	4 weeks
10	*P. eximius*	–		–	–	NT	NT	**+**	4 weeks
11	*P. eximius*	–		–	NT	NT	NT	**+**	1.5 weeks

During breeding, significantly more nest swabs were BFDV-positive in nest boxes which contained BFDV-positive birds (4 out of 11) than in boxes containing BFDV-negative birds (0 out of 11, Fisher’s exact P (one-tailed) = 0.045, P (two-tailed) = 0.09). The difference between nest boxes occupied by BFDV-positive birds and unoccupied boxes was not significant (1 out of 10, Fisher’s exact P (one-tailed) = 0.19, P (two-tailed) = 0.31). Before the breeding season, there was no difference in the number of BFDV-positive nest swabs between the three test groups (Fisher’s exact P_A_ = 1.0, P_B_ = 1.0), as all had BFDV-negative nest swabs. After the breeding season, there was also no difference in the number of BFDV-positive nest swabs (Fisher’s exact P_A_ = 1.0, P_B_ = 0.656), as only one nest with BFDV-positive birds had a BFDV-positive swab, and all other swabs were BFDV-negative. The number of BFDV-positive nest swab samples increased when we shifted the detection threshold of the qPCR to the more sensitive threshold_38_ (Figs. S1B , S2). However, the overall conclusions remained qualitatively the same.

Of the six nests in which we found at least one BFDV-positive parent (*n* = 5 nests of *P. elegans*, *n* = 1 of *P. eximius*), we tested antigen excretion for all female parents. Five of six females (83.3%, 35.9–99.6) had detectable antigen in their feathers, with a mean antigen titre of log_2_3.6 (*n* = 5, 1.04–6.16; Table 1). All of these females had BFDV-positive blood samples (Table 1). Of the *n* = 5 *P. elegans* females with detectable antigen in feathers, *n* = 4 were subadults (<1 year old) and *n* = 1 was a young adult (1 –3 years old). We detected BFDV on nest swabs of four out of five (80.0%, 28.4 –99.5) nest boxes occupied by parental females with antigen excretion (Fig. 2B).

### Duration of BFDV presence in nest boxes

For the one nest which contained a BFDV-positive bird and which had BFDV-positive nest swabs during as well as after breeding (threshold_36_ used for detection, Fig. 2A), the BFDV-positive swab during breeding was collected on October 10, 2016. BFDV-positive blood and cloacal swab samples of the female parent were taken 45 days later, on November 24, 2016, and the BFDV-positive nest swab after the breeding season was collected 68 days after that, on January 31, 2017. The time span between the two positive nest box swabs was 113 days (3.7 months). Using the threshold_38_ for BFDV detection, one more box with a BFDV-positive bird and one unoccupied box had BFDV-positive nest swabs both during and after the breeding season (Fig. S2). The time spans between samplings of these two BFDV-positive nest swabs were 67 days (2.2 months) for the box with the BFDV-positive bird, and 79 days (2.6 months) for the unoccupied box.

### Patterns of infection within nests

We obtained blood samples from all nestlings for all 11 nests which contained a BFDV-positive bird (parent or nestling). We collected blood samples and cloacal swabs of both parents for six of the 11 nests, blood samples from both parents and cloacal swabs from one parent for two of the nests, blood and cloacal samples for only one parent for one nest, and blood samples only for one parent for two nests (Table 1). In the five nests (45.5%, 16.7–76.6) which contained an infected nestling, these nestlings were BFDV-positive only at one of the two age stages we sampled nestlings at: in four out of five cases (80%, 28.4–99.5), the BFDV-positive nestling was approximately four weeks old (Table 1). In three out of these four cases, (75%, 19.4–99.4), all nestlings fledged successfully (Table S1). In the one case where the nestling was 1.5 weeks old when BFDV-positive, the nestling survived and all nestlings in this brood were BFDV-negative at four weeks of age (Table 1, Table S1).

## Discussion

Pathogens which can persist outside of their hosts can potentially play a substantial role in the spread of wildlife disease through indirect transmission via environmental contamination (Rohani et al., 2009). BFDV has been considered an environmentally stable pathogen which is likely to be transmitted indirectly through nest hollows, and is a threat for psittacine birds in Australia and world-wide (Peters et al., 2014). In this first study of BFDV presence in nesting material of wild birds, we found BFDV-infected parents or nestlings in 11 out of 82 (13.4%) nests. Four out of five (80%) boxes with a shedding parent provided a BFDV-positive swab; BFDV-positive nest swabs were also derived from four out of six (66.7%) boxes occupied by parents which were BFDV-positive in blood samples, and in two out of three (66.7%) boxes occupied by parents with BFDV-positive cloacal swabs. Zero out of five (0%) boxes containing BFDV-positive nestlings had BFDV-positive nest swabs. This resulted in a total of four out of 11 (36.4%) boxes occupied by BFDV-positive birds (parents or nestlings) that yielded a BFDV-positive nest swab during the breeding season. This decreased to one out of 11 (9.1%) nests after the breeding season. Using the conservative threshold_36_, both control groups had BFDV-negative nest swabs both before and after breeding.

We found a high percentage (80%) of BFDV-positive nest swabs derived from nests occupied by BFDV-positive females with antigen excretion, and from nests occupied by parents with BFDV-positive cloacal swabs (66.7%), but an absence of BFDV on nest swabs derived from nests with BFDV-positive nestlings. This suggests that results from nest swabs are more strongly associated with shedding of BFDV by infected birds (feathers, cloaca) rather than BFDV presence in blood alone. Antigen excretion into feathers is considered to be an indicator for active BFDV infection with virus replication (Sarker et al., 2016), and contaminated feather dander is thought to be one of the main BFDV transmission routes (Raidal, Sabine & Cross, 1993). More generally, antigen excretion is a major contributor to disease outbreaks originating from environmental contamination, as has been shown in cholera and other diseases (Joh et al., 2009). While we could not test antigen excretion in nestlings or males, all females that were excreting antigen in our study were younger than three years. In *P. elegans*, prevalence is highest in subadult birds (<1 year old) (Eastwood et al., 2014; Martens et al., 2019). Female *P. elegans* start to procreate when they are still subadults, but this is very rarely observed in males (Eastwood et al., 2019; Krebs, 1998). In *P. elegans* and *P. eximius* at least, subadult females may thus play an important role in BFDV transmission in the nest. On the other hand, nest swabs from nests containing BFDV-positive nestlings were all BFDV-negative. In nestlings, BFDV incubation times from exposure to onset of clinical signs can range from 36 h (Doneley, 2003) to four weeks (Raidal & Cross, 1995), and in some cases, nestlings are thought to be protected temporarily by maternal antibodies (Ritchie et al., 1992). Nestlings in our study were possibly not shedding BFDV yet. There are, however, no studies investigating the time span between BFDV infection and onset of shedding. We do not know whether the four nestlings that had tested BFDV-positive at four weeks of age in our study were still BFDV-positive when leaving the nest, and could not test whether they were shedding BFDV.

We show that while BFDV remained detectable in a nest box for up to several months, as predicted in several studies which assumed extended persistence of BFDV outside of the host (Eastwood et al., 2019; Peters et al., 2014; Raidal & Peters, 2018), it was less likely to be detected after the breeding season (only one nest box had a BFDV-positive nest swab after the breeding season). Additionally, we did not close off the nest boxes after the nestlings had fledged. It is therefore possible that BFDV-positive birds accessed the nest boxes after breeding, which may have contributed to nest swabs testing BFDV-positive after the breeding season. As we only tested swabs collected directly after the breeding season, i.e., two to three months after birds occupying the boxes tested BFDV-positive, and not later in the year, we cannot exclude that BFDV can be present for a longer time period than reported here. However, the lack of BFDV detection (threshold_36_) in all boxes (boxes occupied by BFDV-positive birds during the breeding season, and both sets of control boxes) before the breeding season and the very low prevalence of BFDV in nesting material two to three months after the breeding season suggests that at least in our study system, it is unlikely that BFDV persists in the nest box up until the next breeding season. We detected BFDV on nest swabs with a qPCR assay, which is a well-established and very accurate method for the detection of BFDV-infected birds, which detects short fragments of viral DNA (Eastwood et al., 2015). Most qPCR positives, at least those ones derived from blood samples, represent active infection with viable virus as confirmed by sequencing (Eastwood et al., 2014), but some could be non-active remnant viral DNA (Eastwood et al., 2019). As we could not pick up viral DNA fragments with qPCR before or, in most cases, after the breeding season, it seems unlikely that intact, infectious viral particles persist in nest boxes outside of the breeding season. Future studies could consider testing nest swabs with an HA assay (Raidal, Sabine & Cross, 1993), to elucidate whether BFDV-positive results indeed represent contamination of the nest box with viable, infectious antigen.

Despite the low likelihood of detecting BFDV in the nest box after the breeding season, indirect transmission may still occur, just within a shorter time window. Some parrot species inspect nest hollows year-round (Higgins, 1999), and competition over nest hollows is high, particularly in parrots (Heinsohn, Murphy & Legge, 2003; Martens & Woog, 2017). Preventative measures against BFDV may thus still need to be implemented in recovery programs for endangered species. On the other hand, in a recent study by Fogell et al. (2019) on BFDV, hygiene measures did not improve fledging success in an endangered psittacine species. Additionally, a study on avian infections with the bacterium *Mycoplasma gallisepticum* showed that indirect transmission by environmental contamination led to only mild infections, possibly due to exposure to low infectious doses of *Mycoplasma*, resulting in hypothesised immunisation against future infections (Dhondt et al., 2007). Future studies which compare BFDV strains of infected birds in the nest (nestlings, breeding adults), and strains found in the nesting material itself (Eastwood et al., 2019), are needed for a better understanding of transmission routes between birds in the nest, and between nesting material and birds, and the resulting possible positive (immunisation) (Dhondt et al., 2007) or negative (chronic infection) (Peters et al., 2014) effects on hosts.

Overall, BFDV prevalence in breeding birds and their offspring in our study (13.4% of nests, 1.8% of parental blood samples) was much lower than previously reported for *P. elegans* (34.5%) (Eastwood et al., 2015). With one exception where both parents in one nest were BFDV-positive, we only found one BFDV-positive individual (parent or nestling) in each box. Eastwood et al. (2019) found the same when analysing BFDV infection in *P. elegans* parents and their offspring in the nest, and consequently they suggested that infection of nestlings with BFDV-negative parents may have occurred through contaminated nesting material. Alternatively, the BFDV-positive birds in our study may have been infected through ectoparasitic vectors instead of through the nesting material itself, as BFDV has been found in mites on cockatoos (Portas et al., 2017). Exposure to high doses of BFDV is thought to be required to establish infection (Raidal & Peters, 2018). This may explain why in most nests only one bird was BFDV-positive, as viral contamination may not generally be sufficiently high to reach such doses. In populations with higher BFDV prevalence, nesting material could still be an important avenue of transmission.

Although our nest swab results were strongly associated with the infection status of the parents occupying the boxes, particularly in terms of their likelihood of shedding virus, there were some discrepancies. For example, two boxes occupied by BFDV-positive adults with antigen excretion did not have BFDV-positive nest swabs. Such discrepancies may represent limitations of our methodology, for example during sample collection, as not all nesting material in each box was touched with the nest swabs, but only the material in three random locations in the boxes, and BFDV may have thus not been picked up. Alternatively, the inhibitor removal conducted before conducting the lab assays may not have worked on all swabs: Hall et al. (2018) used the same inhibitor removal system as we did in our study, and reported that a very small percentage (<2%) of samples had to be excluded from the study, as not all inhibitors could be removed. In our study, the number of nest swabs that were BFDV-positive varied depending on the qPCR detection threshold chosen. Using the more sensitive threshold, we picked up more weakly BFDV-positive swabs, which resulted in a BFDV-positive nest swab of one nest with BFDV-negative birds before the breeding season, and BFDV-positive nest swabs of two unoccupied control nests after the season. One explanation for this may be that BFDV-positive birds inspected, but did not select, these nest boxes earlier in the season. For example, *P. elegans* often do not choose the same nest box in consecutive seasons, but search for a different nest box (Eastwood et al., 2018; Martens et al., 2018). Alternatively, we cannot exclude the small possibility that this result arose from false positives due to contamination of swabs, as we did not include blank extraction controls in all qPCR assays. We however note that our sampling design, using two types of control nest boxes, repeated sampling of each nest box over three time periods, and basing conclusions on a conservative qPCR threshold (threshold_36_), should reduce concerns about biased results and false positives. Our findings, with all boxes testing BFDV-negative before the breeding season for example, seem logical despite the small possibility of false positives, which gives further reassurance regarding our key results and conclusions. Our comparative results with two different virus detection thresholds show that analysis parameters should be chosen carefully, as they may change the interpretation of results.

## Conclusions

Using nest boxes occupied by BFDV-positive birds, as well as two control groups, we aimed to test whether and for how long BFDV can be detected in nest boxes, and if detection is related to presence of infection with or shedding of BFDV. We had two main goals, namely to test a non-invasive method for pathogen surveillance in the nest, and to shed light on indirect transmission dynamics of a pathogen of global conservation concern. We were able to show that during breeding, BFDV was significantly more likely to be detected in nest boxes of BFDV-positive *P. elegans* and *P. eximius* than in control boxes. We detected BFDV in nest swabs of 80% of boxes occupied by parental females which were excreting BFDV antigen, suggesting that the method we are presenting detects BFDV shedding rather than just infection with BFDV. The method we used is simple, inexpensive and non-invasive, with the potential to detect hosts that are in a state of active BFDV infection with viral shedding. It could potentially be applied to other pathogens which are persistent in the environment, thus aiding pathogen surveillance and preventing the spread of wildlife diseases, reducing risk to biodiversity and human health. Additionally, at least in our study system, we show that BFDV does not seem to be as persistent in the nest as previously thought, suggesting that indirect transmission of this pathogen via contaminated nest boxes may not be likely. Further studies are, however, needed to generalise these findings across other host species and geographic regions.

##  Supplemental Information

10.7717/peerj.9211/supp-1 Supplemental Information 1 Supplemental Figures and Tables

10.7717/peerj.9211/supp-2 Supplemental Information 2 Raw data on BFDV status and breeding success The raw data on BFDV status of nest swabs (derived from qPCR assays for all three test groups of nest boxes. It also contains data on number of eggs, hatching and fledging success for all tested nests containing BFDV positive vs BFDV negative birds.

## Additional Information and Declarations

### Field Study Permissions

The following information was supplied relating to field study approvals (i.e., approving body and any reference numbers): Australian Bird and Bat Banding authority 2319. State of Victoria research permit 10007969.

### Data Availability

The following information was supplied regarding data availability: Data is available in the Supplemental Files.
