# Supplementary material for: A non-invasive method to assess environmental contamination with avian pathogens: beak and feather disease virus (BFDV) detection in nest boxes
Source: PeerJ. 2020 Jun 11;8:e9211. doi: 10.7717/peerj.9211 (PMC7293853; doi:10.7717/peerj.9211)
Supplement: Supplemental Information 1 [file peerj-08-9211-s001.docx]

**Supplementary Material**

**Table S1: Reproductive success in nest boxes containing a BFDV-positive parent or nestling.** ‘Hatching success’ was calculated as percentage of eggs laid that hatched; ‘fledging success’ is percentage of hatched nestlings that left the nest; ‘breeding success’ is percentage of eggs laid that produced fledglings. Shading and order of cells, and numbering of nest boxes, are the same as in Appendix Table 2 (white background indicates nests with BFDV-positive nest swabs; grey background indicates nests with BFDV-negative nest swabs).

| **year** | **nest box** | **species** | **BFDV+**  **nest swab** | **no. eggs**  **laid** | **no.**  **eggs hatched** | **no. fledglings** | **hatching  success (%)** | **fledging  success (%)** | **breeding  success (%)** |
| --- | --- | --- | --- | --- | --- | --- | --- | --- | --- |
| 2016 | 1 | *P. elegans* | + | 6 | 6 | 4 | 100 | 67 | 67 |
| 2016 | 2 | *P. elegans* | + | 7 | 7 | 6 | 100 | 86 | 86 |
| 2017 | 3 | *P. elegans* | + | 6 | 6 | 4 | 100 | 67 | 67 |
| 2017 | 4 | *P. elegans* | + | 6 | 5 | 0 | 83 | 0 | 0 |
| 2016 | 5 | *P. elegans* | - | 6 | 5 | 0 | 83 | 0 | 0 |
| 2016 | 6 | *P. eximius* | - | 5 | 4 | 4 | 80 | 100 | 80 |
| 2016 | 7 | *P. elegans* | - | 7 | 7 | 4 | 100 | 57 | 57 |
| 2017 | 8 | *P. elegans* | - | 6 | 6 | 6 | 100 | 100 | 100 |
| 2017 | 9 | *P. elegans* | - | 4 | 3 | 3 | 75 | 100 | 75 |
| 2017 | 10 | *P. eximius* | - | 6 | 6 | 6 | 100 | 100 | 100 |
| 2017 | 11 | *P. eximius* | - | 6 | 6 | 6 | 100 | 100 | 100 |
|  |  |  | MEAN | 5.9 | 5.6 | 3.9 | 92.9 | 70.6 | 66.5 |
|  |  |  | SD | 0.8 | 1.2 | 2.1 | 9.6 | 36.6 | 34.3 |

**Table S2:** **Data on nest boxes with BFDV-negative birds which we used as control group.** ‘Hatching success’ was calculated as percentage of eggs laid that hatched; ‘fledging success’ is the percentage of hatched nestlings that fledged; ‘breeding success’ is the percentage of eggs laid that produced fledglings. Abbreviations used are: for field sites: Bellbrae (BB), Meredith (ME), Steiglitz (ST) and Gundrys Road (GR); No. (number), BFDV+ (BFDV-positive), blood+ (BFDV-positive blood sample), blood- (BFDV-negative blood sample), cloacal+ (BFDV-positive cloacal swab), cloacal- (BFDV-negative cloacal swab), adult ♀ (female parent), adult ♂ (male parent).

| **year** | **site** | **nest** | **species** | **BFDV+**  **nest swab** | **no. eggs** | **no. eggs hatched** | **no. fledglings** | **hatching  success (%)** | **fledging  success (%)** | **breeding  success (%)** | **no. adults tested** | **BFDV status  adult ♀** | **BFDV status  adult ♂** | **BFDV status  nestlings** |
| --- | --- | --- | --- | --- | --- | --- | --- | --- | --- | --- | --- | --- | --- | --- |
| 2016 | ST | W8 | *P. elegans* | 0 | 9 | 9 | 9 | 100 | 100 | 100 | 2 | blood-, cloacal- | blood-, cloacal- | blood- |
| 2016 | ME | E10 | *P. elegans* | 0 | 6 | 6 | 6 | 100 | 100 | 100 | 2 | blood-, cloacal- | blood-, cloacal- | blood- |
| 2016 | ME | E4 | *P. elegans* | 0 | 6 | 4 | 0 | 67 | 0 | 0 | 2 | blood-, cloacal- | blood-, cloacal- | blood- |
| 2016 | BB | C11 | *P. elegans* | 0 | 5 | 5 | 5 | 100 | 100 | 100 | 2 | blood-, cloacal- | blood-, cloacal- | blood- |
| 2016 | ME | W4 | *P. elegans* | 0 | 6 | 6 | 6 | 100 | 100 | 100 | 2 | blood-, cloacal- | blood-, cloacal- | blood- |
| 2017 | BB | C15 | *P. elegans* | 0 | 7 | 7 | 7 | 100 | 100 | 100 | 2 | blood-, cloacal- | blood-, cloacal- | blood- |
| 2017 | ST | W7 | *P. eximius* | 0 | 6 | 5 | 5 | 83 | 100 | 83 | 2 | blood-, cloacal? | blood-, cloacal? | blood- |
| 2017 | GR | P29 | *P. elegans* | 0 | 6 | 4 | 4 | 67 | 100 | 67 | 2 | blood-, cloacal- | blood-, cloacal- | blood- |
| 2017 | ST | S16 | *P. elegans* | 0 | 4 | 3 | 3 | 75 | 100 | 75 | 2 | blood-, cloacal- | blood-, cloacal- | blood- |
| 2017 | ST | S20 | *P. elegans* | 0 | 6 | 6 | 6 | 100 | 100 | 100 | 2 | blood-, cloacal- | blood-, cloacal- | blood- |
| 2017 | BB | N7 | *P. elegans* | 0 | 6 | 4 | 3 | 67 | 75 | 50 | 2 | blood-, cloacal- | blood-, cloacal- | blood- |
|  |  |  |  | MEAN | 6.09 | 5.36 | 4.91 | 87.12 | 88.64 | 79.55 |  |  |  |  |
|  |  |  |  | SD | 1.16 | 1.61 | 2.27 | 14.81 | 28.93 | 30.02 |  |  |  |  |


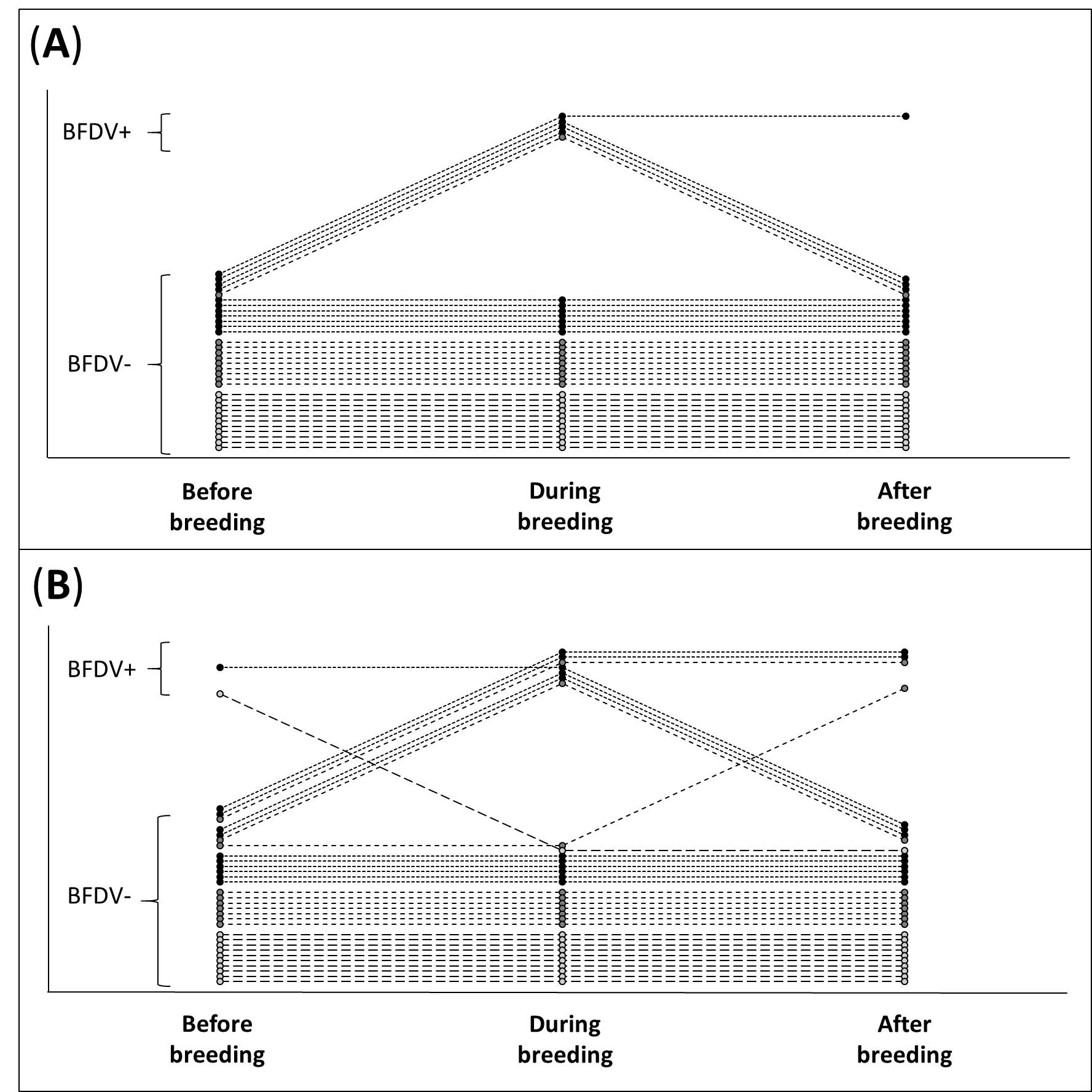


**Figure S1: BFDV presence in the nest box before, during and after the breeding season for every nest, as detected with nest box swabs.** Panel A shows results with a detection threshold of Cq < 36, panel B shows the same nest, with a detection threshold of Cq < 38. Black dots with dotted lines show nests which contained BFDV-positive birds, dark grey dots with short-dashed lines show empty nest boxes which were used as paired controls, and light grey dots with long-dashed lines show nest boxes with BFDV-negative birds. Dots have been spread out along the y-axis for better visibility.

**
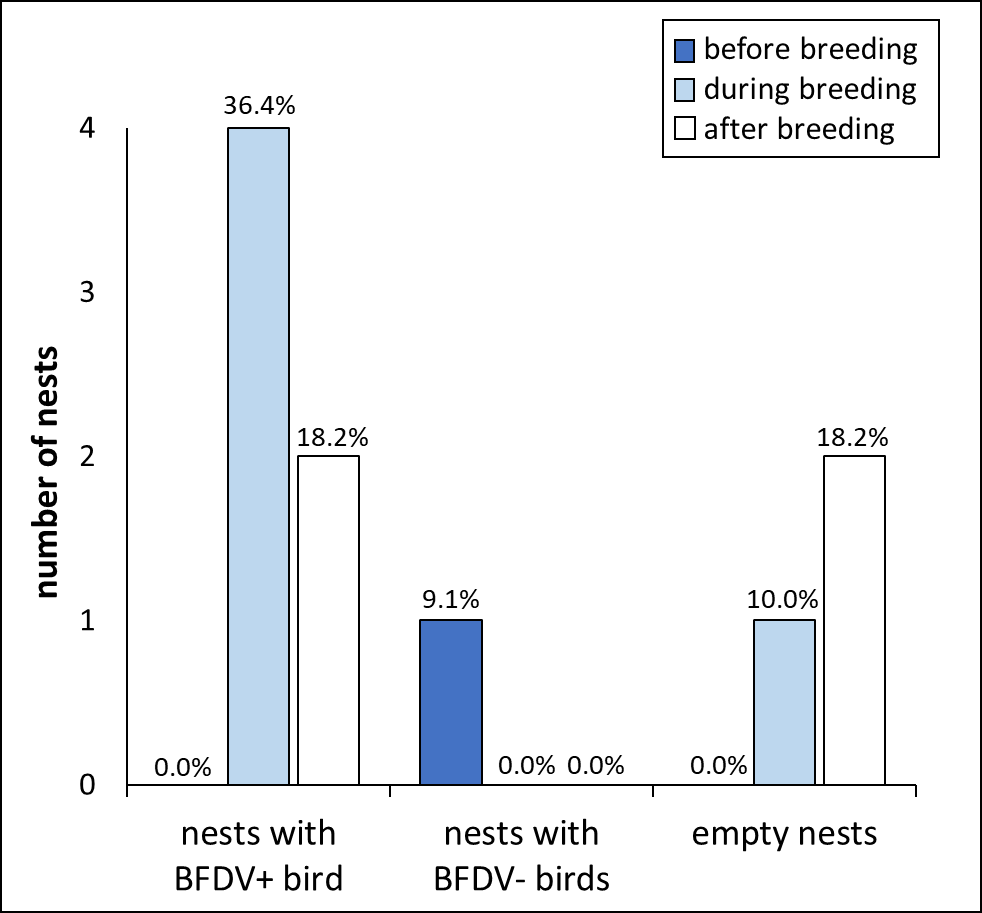
**

**Figure S2:** **Number of nest boxes (out of total of 33 for the three test groups, 11 per group) with BFDV-positive nest box swabs before, during and after the breeding season, and analysed with the higher qPCR threshold_38_.** Using this threshold leads to inclusion of samples with weaker qPCR signals as positive and thus to a higher total number of BFDV-positive samples. For unoccupied nest boxes during the breeding season, the sample size was 10 unoccupied nest boxes instead of 11.
